# Metal ion coordination in the *E*. *coli* Nudix hydrolase dihydroneopterin triphosphate pyrophosphatase: New clues into catalytic mechanism

**DOI:** 10.1371/journal.pone.0180241

**Published:** 2017-07-25

**Authors:** Shannon E. Hill, Elaine Nguyen, Chiamaka U. Ukachukwu, Dana M. Freeman, Stephen Quirk, Raquel L. Lieberman

**Affiliations:** 1 School of Chemistry and Biochemistry, Georgia Institute of Technology, Atlanta, Georgia, United States of America; 2 Kimberly-Clark Corporation, Roswell, Georgia, United States of America; Yale University School of Medicine, UNITED STATES

## Abstract

Dihydroneopterin triphosphate pyrophosphatase (DHNTPase), a member of the Mg^2+^ dependent Nudix hydrolase superfamily, is the recently-discovered enzyme that functions in the second step of the pterin branch of the folate biosynthetic pathway in *E*. *coli*. DHNTPase is of interest because inhibition of enzymes in bacterial folate biosynthetic pathways is a strategy for antibiotic development. We determined crystal structures of DHNTPase with and without activating, Mg^2+^-mimicking metals Co^2+^ and Ni^2+^. Four metal ions, identified by anomalous scattering, and stoichiometrically confirmed in solution by isothermal titration calorimetry, are held in place by Glu56 and Glu60 within the Nudix sequence motif, Glu117, waters, and a sulfate ion, of which the latter is further stabilized by a salt bridge with Lys7. *In silico* docking of the DHNTP substrate reveals a binding mode in which the pterin ring moiety is nestled in a largely hydrophobic pocket, the β-phosphate activated for nucleophilic attack overlays with the crystallographic sulfate and is in line with an activated water molecule, and remaining phosphate groups are stabilized by all four identified metal ions. The structures and binding data provide new details regarding DHNTPase metal requirements, mechanism, and suggest a strategy for efficient inhibition.

## Introduction

Enzymes involved in folate biosynthesis in bacteria are targets of antimicrobial agents that have been used in the clinic since the 1930s [1, 2]. The development of new folate-like antimicrobials has been aided by the availability of genes encoding these enzymes and emergence of new biochemical and structural details. One addition to the *E*. *coli* pathway, dihydroneopterin triphosphate pyrophosphatase (DHNTPase), was first isolated in 1974 [3] but its biological role in folate biosynthesis was confirmed only 30 years later [4, 5]. DHNTPase catalyzes the hydrolysis of 7,8-dihydroneopterin triphosphate, the product of GTP cyclohydrolase, to 7,8-dihydroneopterin monophosphate and pyrophosphate [4]. DHNTPase can now be included in an antibiotic strategy that, like classical sulfa-class antibiotics [1], simultaneously targets multiple enzymes in the folate biosynthetic pathway.

DHNTPase belongs to the Nudix hydrolase superfamily (InterPro IPR000086; Pfam PF00293), which catalyze the hydrolysis of a variety of phosphorylated nucleotide molecules and their derivatives, as well as some non-nucleotide substrates like DHNTP [6]. These enzymes are found in a broad spectrum of organisms and their physiological role is generally thought to be in the recycling of metabolic products in order to control their cellular concentration [7]. These family members share a so-called Nudix motif (Prosite PS00893) GxxxxxExxxxx[UA]xRExxEExG, where x is any amino acid and U is an aliphatic hydrophobic residue. The motif RExxEE binds a required metal ion cofactor, presumed physiologically to be Mg^2+^. Substrate specificity is dictated outside of this motif in respective enzymes. In spite of the prevalence of this signature motif and similar reaction mechanism, Nudix hydrolases appear to differ in the catalytic base responsible for activating the water for hydrolysis and the number of metal ions involved in catalysis [6].

To better understand how DHNTPase catalyzes its reaction and drive drug discovery efforts, we solved crystal structures of apo, Co^2+^-, and Ni^2+^- bound DHNTPase in a new crystal form. The metallated structures reveal a tetranuclear metal binding site coordinated by enzyme residues within and outside of the Nudix signature motif, water molecules, and a sulfate anion used during crystallization. DHNTPase is active in the presence of these octahedral-coordinating transition metals, and binding of four metal ions in the presence of sulfate was confirmed by isothermal titration calorimetry. *In silico* docking of the substrate reveals that the crystallographic sulfate is in the position of the β-phosphate, which is rendered electrophilic by its myriad interactions. A solvent molecule, positioned in line with the β-phosphate, appears activated for nucleophilic attack by three of the four metal ions. Here we describe in detail these structures as well as insights into implications for catalysis and inhibition.

## Materials and methods

### Cloning, expression, and purification

Genomic DNA from a laboratory strain of *E*. *coli* K12 was isolated using a QIAGEN genomic DNA isolation kit according to the manufacturer’s directions. Primers were designed to incorporate a Nde I and Bam HI restriction site just prior to the initiating ATG codon and just after the final TAA stop codon, respectively, and were of the following sequence: 5’-ATATATCATATGTTGCAGCGGCGTGAC (5’ primer) and 5’-ATATATGGATCCTTAATTACAAACTGT (3’ primer). Amplified DNA was purified via a QIAGEN PCR purification procedure and was digested with Bam HI and Nde I at 37°C for an hour. Digestion product was directly added to similarly digested pET11d yielding a non-cleavable His-tag DHNTPase construct.

To produce an N-terminal cleavable His-tag DHNTPase construct, DHNTPase was subcloned into pET-30 Xa/LIC vector (Novagen) following the manufacturer’s directions. Primers were designed to incorporate a final TAA stop codon and were of the following sequence: 5’- GGTATTGAGGGTCGCAAAGATAAAGTTTACAAACGTCCGG (5’ primer) and 5’- AGAGGAGAGTTAGAGCCTTACGCAGCATTGATGACGAATTGTTCGATCGCTTGGCGG (3’ primer). Amplified DNA was purified via a Promega PCR purification procedure, treated with T4 DNA Polymerase, and annealed into the pet 30-xa/LIC vector. An aliquot was used to transform competent *E*. *coli* NovaBlue GigaSingles. Plasmid fidelity was confirmed by DNA sequencing (MWG Operon).

DHNTPase was expressed in *E*. *coli* BL21 (DE3) cells. A single transformed colony was inoculated into a selective 400 mL Luria-Bertani culture (LB, Fisher, supplemented with 50 ug/mL kanamycin) and agitated at 220 r.p.m., 37°C, overnight (16–24 hours). A starter culture (30 mL) was used to inoculate 1L of LB media, which was then agitated at 220 r.p.m., 37°C until an optical density at 600 nm of 3.0–3.5 was reached. Protein expression was induced with 0.5 mM ispropyl β-D-thiogalactopyranoside (IPTG) and induced cells were grown overnight (16–20 hours) by agitation at 220 r.p.m., 18°C. Cells were harvested by centrifugation and flash frozen in liquid nitrogen for storage at -80°C.

Cell pellets (5 g) were suspended into 50mL Nickel affinity purification wash buffer (50 mM Hepes pH 7.5, 500 mM NaCl, 20 mM imidazole) and homogenized using a serological pipette followed by 2–3 passages through a French press (12,000 psi) to lyse the cells. Cellular debris was removed by ultracentrifugation at 130,000 × g, 4°C for 45 minutes and the supernatant loaded onto a 1 mL Nickel affinity purification column (GE Healthcare) using an AKTA purification system (GE Healthcare). Wash buffer was then applied to remove contaminating proteins, followed by an elution gradient from 20-300mM imidazole to isolate pure DHNTPase. Fractions containing DHNTPase were concentrated and buffer exchanged three times into Factor Xa cleavage buffer (50mM Tris pH 7.5, 1mM EDTA) using an Amicon 10K MWCO filter (EMD Millipore). To remove N-terminal tags DHNTPase was diluted to a final concentration of 1 mg/ml in Factor Xa cleavage buffer and incubated with Factor Xa (Roche, 50:1 mass ratio) for 20–24 hours at room temperature. The digested DHNTPase was concentrated in an Amicon 3K MWCO filter (EMD Milipore) to 1mL and loaded onto a HiLoad 16/600 Superdex 75 prep grade column (GE Healthcare) equilibrated with gel filtration buffer (50 mM Tris pH 7.5, 1mM EDTA). The fractions containing pure DHNTPase were assessed by 15% SDS-PAGE analysis with Coomassie staining and concentrated in an Amicon 3K MWCO filter. Protein concentration was measured by absorbance at 280 nm using a molecular extinction coefficient (28,085 M^-1^cm^-1^) and a molecular weight (17,174.5 g/mol) calculated by ExPASy ProtParam [8].

### Crystallization and structure determination

Crystallization conditions were identified by sparse matrix screening of cleaved DHTNPase and subsequently refined for concentration of PEG, ammonium sulfate, buffer and temperature. Crystals of DHNTPase grew after approximately three days. A well solution containing 35% PEG 8000, 0.05 M ammonium sulfate was mixed 1:1 with 20 mg/ml DHNTPase in 50 mM Tris pH 7.5, 1 mM EDTA pH 7.4 (2 μl total) in a sitting drop configuration and allowed to equilibrate at room temperature. Crystals of Co-bound DHNTPase were soaked in 6 μl of a solution containing mother liquor, 0.5 mM DHNT, 5 mM CoCl_2_•6H_2_O for ~68 h. Crystals of Ni-metallated DHNTPase were soaked similarly but with 0.5 mM DHNT, 10 mM NiCl_2_•6H_2_O, for ~92 h. Crystals of apo DHNTPase were obtained after soaking crystals as above in 0.5 mM DHNT, 10 mM MgSO_4_ for ~92 h.

Data for metallated DHNTPase were collected at the SER-CAT beamline 22-ID at the Advanced Photon Source Argonne, IL and processed with XDS [9]. The Co-anomalous data were collected at an X-ray beam wavelength of 1.6063 Å, and Ni-anomalous at 1.4938 Å. Data for Mg-soaked DHNTPase were collected at SER-CAT beamline 22-BM using a 1 Å beam and processed with HKL2000 [10]. Crystals were solved by molecular replacement using PDB code 2O1C (Chain A) and refined using Phenix.refine with metal ligand restraints obtained by ReadySet within Phenix [11]. Anomalous maps were generated using Phenix.maps within Phenix. The structures were submitted to the PDB with codes 5U7E, 5U7F, 5U7H.

### *In silico* docking and structural analysis

The structure of dihydroneopterin triphosphate was drawn in ChemDraw Professional 15.1 (Perkin Elmer) and saved in MOL3d format. Elbow [12] within Phenix was then used to obtain a PDB file with charges and restraints. The protein was prepared by the use of the PDB2PQR server [13]. The selected forcefield was PARSE and pH value was set to 7.4. AutoDock Vina within the PyMOL plugin [14] was used for docking. The grid was set generously to encompass the complete active site, and the ligand was allowed to be flexible. The top 10 docked molecules from both the apo structure and Co-bound structure (without heteroatoms) were manually inspected and compared. The preferred pose described was one most compatible with the position of the ring structure, crystallographic sulfate, water, and metal ions.

The DALI server [15] was used to identify the closest structural homologs to DHNTPase. Sequence alignments were conducted using PROMALS3d [16] and prepared in the figure by ESPript [17]. Structure superimpositions were conducted using the secondary structure matching [18] algorithm within Coot [19]. Figures depicting structures were prepared in PyMOL.

### Enzyme assays

The enzymatic activity of DHNTPase was measured by detecting the release of pyrophosphate from ATP via conversion to orthophosphate using a colorimetric ATPase/GTPase Activity Kit (Sigma MAK113 or US Biological 298996). For metal-specific enzymatic activity assays, cleaved DHNTPase was first loaded onto a HiLoad 16/600 Superdex 75 prep grade column (GE Healthcare) equilibrated with 50mM Hepes pH 7.5 buffer to remove traces of EDTA. DHNTPase (final concentration 73 μM) was mixed with 1 mM ATP supplemented with either no added metal ions, 5mM MgCl_2_•6H_2_O, 5mM NiCl_2_, or 5mM CoCl_2_•6H_2_O in 25mM Hepes pH 7.5. For Mg^2+^ concentration-dependence assays (US Biological 298996 kit) DHNTPase (final concentration 64 μM with 0.25 mM EDTA) was mixed with 1mM ATP supplemented with no 0, 2, 4, or 5 mM MgCl_2_•6H_2_O in assay buffer containing 20 mM Tris pH 7.5, 40 mM NaCl, 0.5 mM EDTA. Ratios of metal-to-enzyme were estimated by taking into account chelation by EDTA in relevant solutions. For both variants of the enzyme, the reaction time was 30 minutes at room temperature followed by adding malachite green, which quenches the reaction, and waiting another 30 minutes. Liberated phosphate was measured by absorbance at 620 nm and compared to a standard curve (0–50 μM free phosphate) recommended by the manufacturer. The amount of DHNTPase added to the reaction was constrained to remain within the linear region of this standard curve after subtraction of reactions with no DHNTPase. For metal specific activity assays, final values reported are an average of two independent measurements with three samples for each reaction condition. For dose-dependent metal activity assays, the final values reported are an average of three samples for each reaction condition.

### Isothermal titration calorimetry (ITC)

ITC was performed with a VP-ITC instrument (MicroCal, Inc) using cleaved DHNTPase. Titrations were carried out by injecting 5 μL of a CoCl_2_•6H_2_O or NiCl_2_•6H_2_O solution (0.5–2.0 mM) into the 1.4 mL stirred reaction cell. DHNTPase was varied from 50 to 80 *μ*M in the cell. Both the metal salt and the enzyme were prepared in 20 mM sodium cacodylate (pH 7.0), 40 mM NaCl, and 100 μM sodium sulfate. Titrations were conducted at 25°C. Typical experimental conditions for the titrations were a 10 s injection period followed by a 240 s delay between injections for a total of 40 injections. Blank titrations of metal into buffer were performed to correct for heats of dilution and mixing. The independent set of multiple binding sites is the most common model for binding experiment evaluations. The analytical solution for the total heat used in this study was as described by Freire *et al*.[20]:

$$Q=V\Delta H\left[[L]+\frac{1+[P]nK-\sqrt{{\left(1+[P]nK-[L]K\right)}^{2}+4K[L]}}{2K}\right]$$

where Q is the total heat, V is the cell volume, ΔH is the enthalpy, P is the DHNTPase concentration in the cell, n is the binding stoichiometry, L is the ligand concentration (the binding partner in the syringe, Ni^2+^ or Co^2+^), and K is the association constant. Data were fit to this model using Origin version 7 (MicroCal, Inc.). Gibbs free energy was then calculated according to the relationship ΔG = ΔH-TΔS.

The model of sulfate binding to DHNTPase could not be reconciled with the simple binding model used for metal binding to the DHNTPase /SO_4_^2-^ complex. Instead, data could only be fit to a sequential binding model for the case where n = 2 in the form

$$Q=[P]V\;\left[{\Delta H}_{1}(\frac{K_{1}[L]}{1+K_{1}[L]+K_{1}K_{2}{\left[L\right]}^{2}})+(\Delta H_{1}+\Delta H_{2})(\frac{K_{1}K_{2}[L]}{1+K_{1}[L]+K_{1}K_{2}{\left[L\right]}^{2}})\right]$$

where the terms and fitting are as above.

### Circular dichroism spectra and melts

Protein was diluted to a final concentration of 20 μM in 15 mM phosphate buffered saline pH 7.0 for far UV (195–250 nm) measurements. Spectra were acquired as a function of temperature on an Applied Photophysics Chirascan spectrophotometer utilizing a 10 mm pathlength quartz cuvette. Melting curves were obtained at 1°C intervals after a 5 min incubation at the new temperature with an averaging time of 5 seconds. Sulfate (50 μM) and metal (100 μM) were added to the buffer/protein mix for the scans of sulfate or sulfate/metal DHNTPase complexes. Thermal denaturation was fully reversible as evidenced by recovering ~96% of the CD signal upon cooling and by the observation that reverse and forward melting curves were superimposable.

CD signal at 217 nm were fit to a 2-state equilibrium unfolding model after a linear fit of the folded and unfolded baselines according to:

$$F_{u}=\frac{\theta_{f}-\theta_{i}}{\theta_{f}-\theta_{u}}$$

where F_u_ is the fraction folded, *θ*_*f*_, *θ*_*u*_, and *θ*_*i*_ are the CD signal of the native, unfolded, and i^th^ temperature state respectively. Finally, the data presented represent the average of three independent experiments, all of which were superimposable to within 0.25°C of T_m_.

## Results and discussion

### Overall structure comparison

Initially, we sought to crystallize DHNTPase in the presence of dihydroneopterin as a first step toward inhibitor design. Unlike its phosphorylated counterpart, dihydroneopterin was commercially available. As part of this process, we identified new PEG-based crystallization conditions for DHNTPase, including one containing a salt cocktail of 20 mM Ni^2+^, Mg^2+^, and Fe^3+^. Optimized crystals grown in ~35% PEG 8000, 0.05 mM ammonium sulfate were soaked in a range of dihydroneopterin concentrations and either 10 mM MgSO_4_, NiCl_2_, or 5 mM CoCl_2_. We solved the respective three ~2 Å resolution structures in space group C2 containing one monomer per asymmetric unit (Table 1). None of the structures contained bound dihydroneopterin, but those soaked with transition metals contained bound metal ions mediating the interaction between DHNTPase and a sulfate anion (see below).

**Table 1:** Data collection and refinement statistics.

	DHNTPase	DHNTPase with Ni^2+^	DHNTPase with Co^2+^
Wavelength (Å)	1.000	1.486	1.606
Resolution range (Å)	29.07–1.94 (2.01–1.94)	33.55–2.00 (2.07–2.00)	57.20–1.79 (1.85–1.79)
Space group	C 1 2 1	C 1 2 1	C 1 2 1
Unit cell (dimensions, Å) (angles, °)	53.32 42.91 56.95 90 91.8 90	53.68 42.98 57.05 90 91.36 90	54.6 43.09 57.23 90 91.87 90
Total reflections	56446 (4861)	59928 (5845)	83327 (3654)
Unique reflections	8915 (888)	8794 (845)	12037 (941)
Multiplicity	6.3 (6.1)	6.8 (6.9)	6.9 (3.9)
Completeness (%)	92.57 (93.30)	98.65 (97.02)	94.84 (75.16)
Mean I/sigma(I)	12.81 (3.35)	11.56 (4.14)	12.61 (2.82)
Wilson B-factor (Å^2^)	25.63	20.10	20.65
R-merge	0.125 (0.593)	0.1764 (0.5763)	0.1352 (0.5547)
R-meas	0.137	0.1914	0.146
CC_1/2_	0.993 (0.871)	0.991 (0.917)	0.991 (0.866)
CC*	0.998 (0.965)	0.998 (0.978)	0.998 (0.963)
R-work	0.1952 (0.2388)	0.1800 (0.2511)	0.1771 (0.3372)
R-free	0.2382 (0.2537)	0.2487 (0.3126)	0.2402 (0.4132)
Number of non-hydrogen atoms	1342	1323	1323
macromolecules	1201	1175	1175
metal ion	N/A	5	4
sulfate	5	15	10
glycerol	6	N/A	N/A
water	130	129	134
Protein residues	144	144	144
RMS (bonds, Å)	0.002	0.007	0.011
RMS (angles, °)	0.43	0.76	1.26
Ramachandran favored (%)	99	98.6	99
Ramachandran allowed (%)	1.0	1.4	1
Ramachandran outliers (%)	0	0	0
Clashscore	4.69	4.67	3.41
Average B-factor (Å^2^)	28.03	25.04	26.70
macromolecules	27.40	24.47	26.00
metal ion	N/A	29.65	26.12
sulfate	23.94	29.02	23.05
glycerol	55.81	N/A	N/A
solvent	32.76	29.63	33.20

Statistics for the highest-resolution shell are shown in parentheses. 10% of reflections were reserved for R-free calculations. N/A, not applicable.

**Fig 1:**
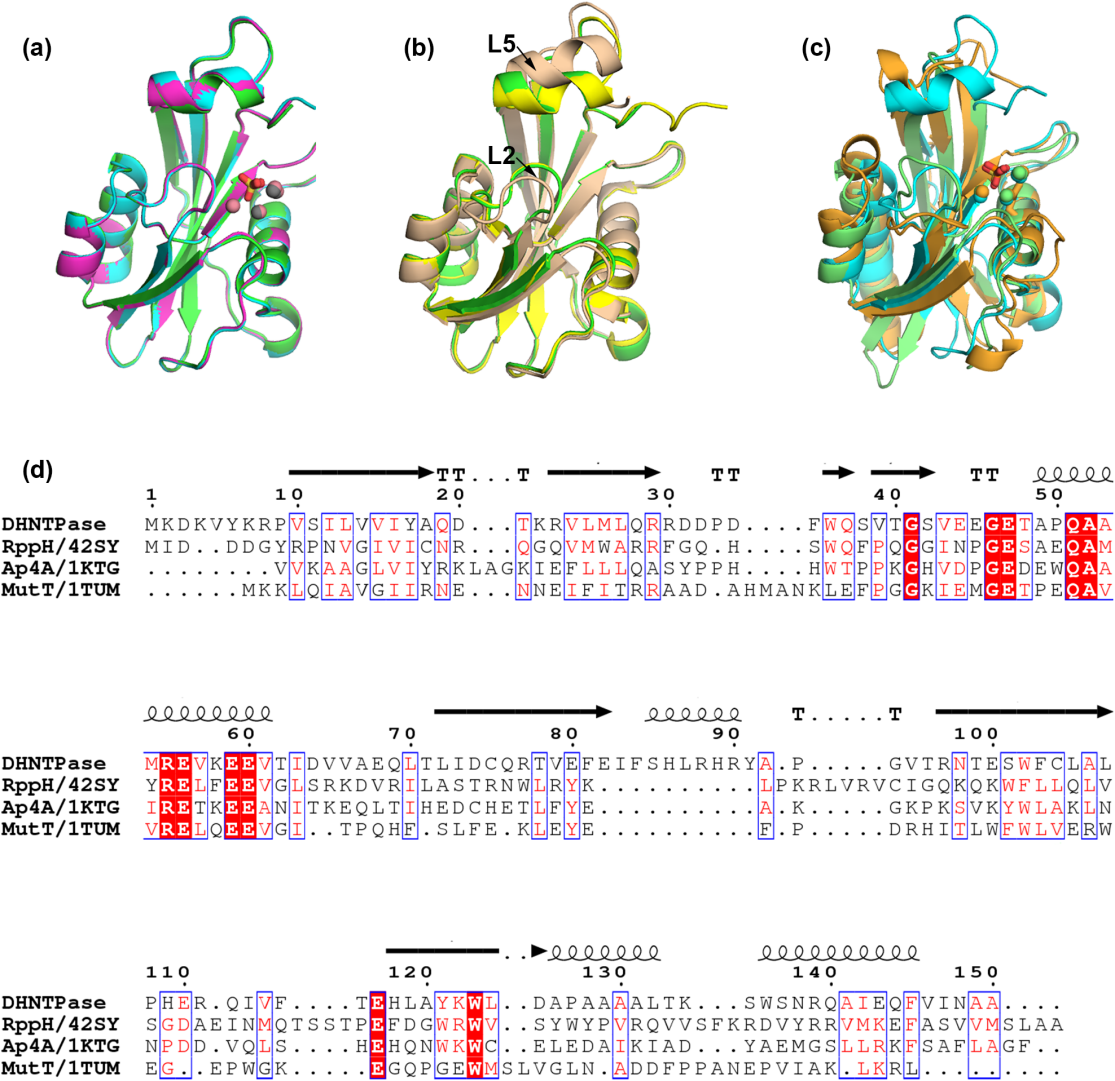
Overall structure and sequence comparison among DHNTPase structures and other Nudix hydrolases. (a) Superposition of apo DHNTPase (green), Ni-bound (magenta), and Co-bound (blue) structures from this study. Sulfates are presented as sticks, and balls represent metals. (b) Superposition of apo DHNTPase with two distinct monomers from PDB code 201C (yellow, beige). (c) Superposition of Co-bound structure (blue) with close structural homologs 1KTG (light green) and 4S2Y (orange). Sulfate from Co-bound structure and phosphate from 1KTG are presented as sticks, and balls represent metals. (d) Multiple sequence alignment of DHNTPase with representative Nudix hydrolases RppH, Ap4A, and MutT. Similar residues are boxed and red font. Identical residues are white with red background. Secondary structure of DHNTPase presented on top of the sequence. β-strands: arrows, helices: coils, T: turns.

The polypeptide chains for all three variations overlay well with each other (Fig 1A, root mean squared deviation (r.m.s.d.) < 0.3 Å) and previous structures of DHNTPase solved using a different crystallization condition and space group, with four molecules in the asymmetric unit (Fig 1B, r.m.s.d. 0.3–1.3 Å) [4]. The main difference between our structures and those previously reported is that there is no shift in loops labeled L2 and L5 (Fig 1B), which was proposed to be a conformational change needed for catalysis [4]. In the analysis below, we focus on the Co-bound structure, which was refined to the highest resolution.

Comparative analysis of our DHNTPase structures with others in the PDB reveals that the two closest structural homologs (~3 Å r.m.s.d.) are also Nudix hydrolases, substrates of which are diadenosine tetraphosphate (*C*. *elegans* Ap4A, PDB code 1KTG)[21] and RNA (*E*. *coli* RppH, PDB code 4S2Y) [22] (Fig 1C). As expected in this family of enzymes, tertiary structure is conserved although overall sequence homology among DHNTPase, Ap4A, RppH, and the prototype Nudix hydrolase MutT [7], is only ~25% (Fig 1D).

### Crystallographic characterization of metal ion-sulfate cluster

**Fig 2:**
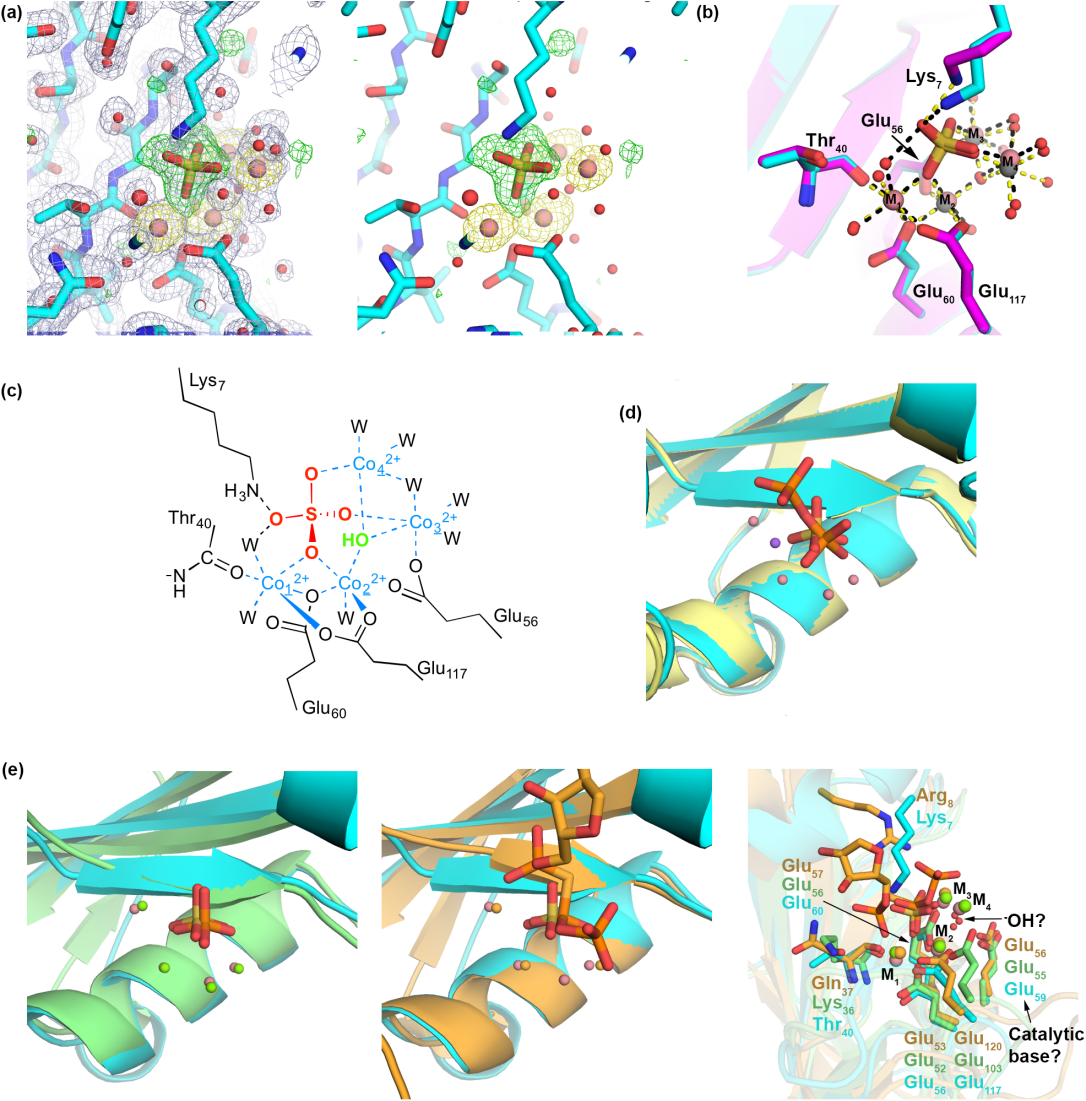
DHNTPase metal cluster. (a) Left: Final 2Fo-Fc electron density map (blue) for Co-bound DHNTPase contoured at 1 sigma superimposed with Fo-Fc map (green) contoured at 3 σ (generated by using only the final polypeptide chain) and an anomalous Fourier map (yellow) contoured at 5 σ. Right: Fo-Fc and anomalous maps only. (b) Superposition of Ni-bound DHNTPase (magenta) and Co-bound DHNTPase (cyan) zoomed into the metal binding site. Dashed lines are selected interactions ≤2.5 Å from protein, sulfate, and/or metal. (c) Chemdraw representation of all ~2.5 Å interactions observed crystallographically in the Co^2+^-bound structure. Numbered metals are referenced in text. W = water. (d) Superposition of DHNTPase solved previously with PPi (orange stick) and Na^+^ (purple ball) from PDB code 2O5W chain A (pale yellow cartoon) and Co-bound DHNTPase (cyan) zoomed into metal centers. (e) Left: Superposition of Ap4A (light green) and Co-bound DHNTPase (cyan) zoomed into metal centers. Both structures contain four metals and a bound sulfate or phosphate. Middle: Comparison of metal binding region of RppH (orange) and Co-bound DHNTPase. RppH was crystallized with three metals overlapping with M1-3 in our structure; the DHNTPase sulfate overlays well with the pyrophosphate leaving group of the cocrystallized substrate analog in RppH. Right: Superposition of all three structures highlighting key coordinating residues to metal and substrate. All three structures have a modeled solvent molecule in line with a putative catalytic base. A unique feature of DHNTPase in this region is involvement of Lys7 at the N-terminus.

Although crystals soaked with Mg^2+^ contained only water molecules in the active site (not shown), those soaked with Co^2+^ or Ni^2^ appeared to be metallated. The active site contents of these crystals were confirmed by datasets collected at the respective anomalous edge. The refined Ni^2+^- and Co^2+^- bound DHNTPase structures are nearly indistinguishable (r.m.s.d. 0.16 Å). Four metal ions (labeled M1-4) are present in each active site, with the remaining electron density attributable to a phosphate-simulating sulfate anion from the crystallographic solution (Fig 2A and 2B). In the Ni^2+^ bound structure, a fifth Ni^2+^ appeared fortuitously bound to solvent-exposed His107 over 30 Å from the active site (not shown). Ni^2+^ and Co^2+^ are excellent mimics of Mg^2+^ binding as they have similar cationic radii, coordination numbers, and preferences for coordination geometry [23, 24].

The Ni^2+^ and Co^2+^ ions are held in place by the same combination of side chains from the protein molecules, solvent, and the sulfate anion (Fig 2B and 2C). M1 is coordinated to an oxygen of the sulfate anion that also bridges to M2, Glu60 within the Nudix signature sequence, the main chain carbonyl from Thr40 (just N-terminal to the start of the Nudix motif), Glu117, and two water molecules, one of which is also within hydrogen bonding distance with a second oxygen from the sulfate anion. The remaining three metal ions form a near-isosceles triangular arrangement (~3.5, ~3.5, 3.1 Å), each ~2.2(Co)/2.0 Å(Ni) away from respective oxygens from the sulfate. M2 shares coordination to the same sulfate oxygen, Glu60, and Glu117 as M1, Glu56 of the Nudix motif, one water molecule, and a likely deprotonated solvent shared among M2-M4 (see below). M3 is held in place by a sulfate oxygen, Glu56, the aforementioned solvent molecule, and three additional water molecules, one of which bridges to M4. In addition to the bridging solvent, M4 coordinates two other water molecules, and sulfate oxygen, but no atoms from DHNTPase. Viewed from the vantage point of the sulfate anion, four metal ions coordinate three of the four oxygen atoms; Lys7 from DHNTPase is 2.4 Å(Co)/2.5 Å(Ni) away from the fourth sulfate oxygen, likely forming a salt bridge.

Catalytically-relevant metal ions were not present in previously solved structures of DHNTPase. A single Sm^3+^ was used to obtain experimental phases, and found proximal to our M2. In crystals soaked with pyrophosphate (PPi), a single Na^+^ ion, located between M2 and M3 in our structures, stabilizes the PPi, which is otherwise held in place by numerous protein side chains. The position of our sulfate overlays well with the more solvent exposed phosphate of the PPi in these structures (Fig 2D).

The positions of our metals match those found in structural homologs Ap4A and RppH, which have been crystallized with four [21, 25] and three [22, 26] Mg^2+^ ions in their active site (Fig 2E). M1-M3 is represented in all three structures; RppH does not have a modeled metal ion in position of our M4, the metal ion that does not come in contact with protein residues. Coordination environments among the three enzymes are also very similar: All three glutamates involved in metal ligation in DHNTPase overlay well with corresponding Glu residues in Ap4A and RppH (Fig 2E). Coordination of M1 via a main chain carbonyl at the equivalent position of Thr40 is also retained, but the side chain is not conserved. The unique feature of DHNTPase compared to its closest structural homologs is its N-terminal residue stretch. Ap4A and RppH do not have an analogous interaction to that of Lys7 in DHNTPase; in RppH, Arg8 is proximal to Lys7, but not to substrate/product. In sum, the high level of similarity in metal binding in our metallated DHNTPase structures with Ap4A and RppH indicate that DHNTPase functions with a similar metal requirement that could not be inferred from previous studies.

### Confirmation of tetranuclear metal ion-sulfate cluster binding to DHNTPase in solution

**Fig 3:**
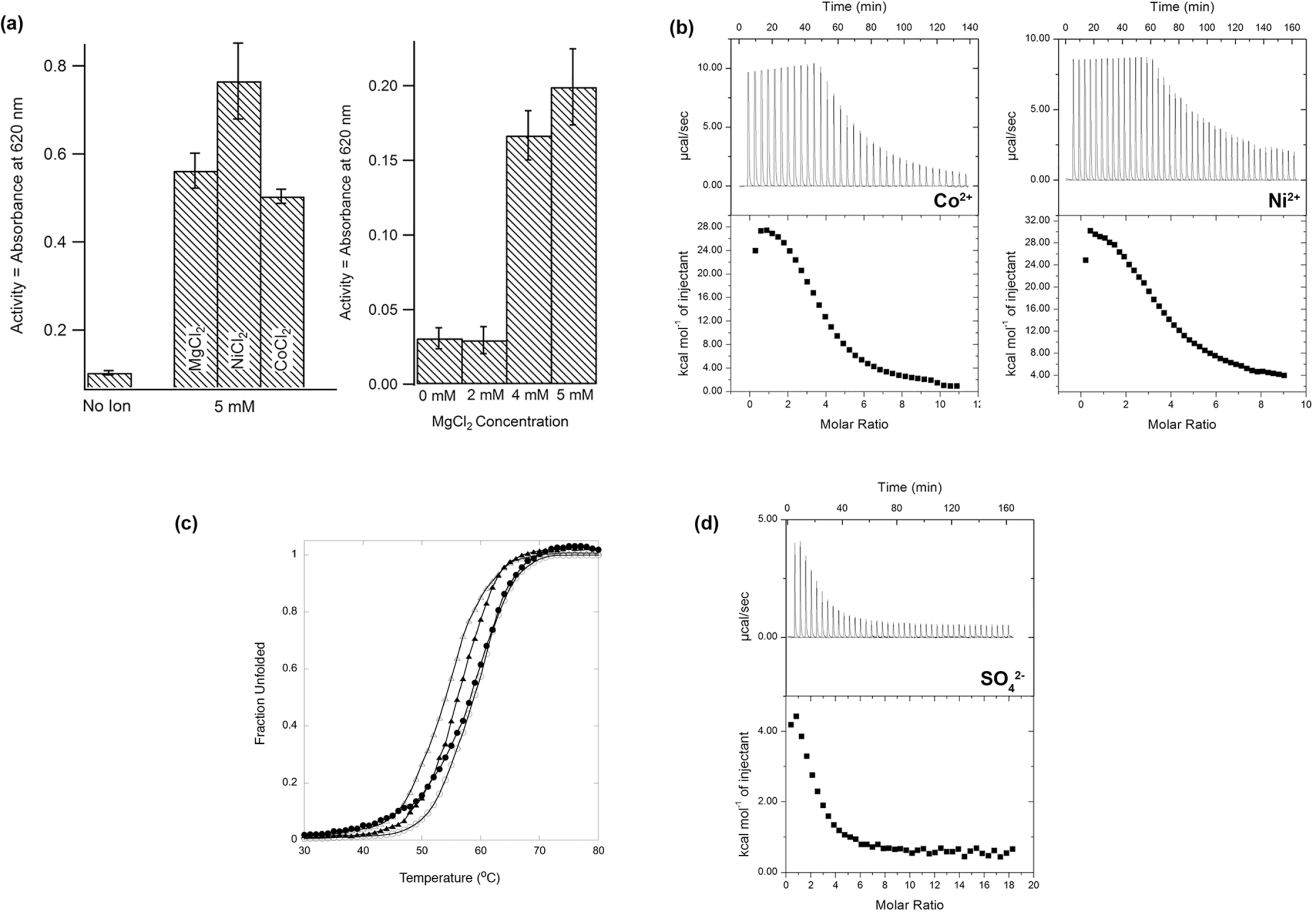
Biochemical characterization of metal binding to DHNTPase. (a) Enzyme assays with varying metals (left) and dose dependence with increasing concentrations of Mg^2+^. (b) Typical ITC binding analysis between DHNTPase and Co^2+^ (left) and Ni^2+^ (right). (c) Fraction unfolded DHNTPase as a function of temperature. Thermal CD scans were performed as described in Methods. Open triangles, Wild-type apo DHNTPase; Closed circles, DHNTPase/SO_4_^2-^/Ni^2+^; Open circles, DHNTPase/SO_4_^2-^/Co^2+^; Closed triangles, DHNTPase/SO_4_^2-^. (d) Typical ITC binding analysis between apo DHNTPase and SO_4_^2-^. Thermodynamic parameters for ITC experiments in (b) and (d) are listed in Table 4.

In support of the biological relevance of the crystallographic observation of four metal ions beyond ionic radii and coordination preferences, we conducted enzyme activity assays with a colorimetric assay using ATP, identified previously and a convenient proxy for the natural substrate DHNTP, which is not available commercially [27]. DHNTPase is active when either Co^2+^ or Ni^2+^ replaces Mg^2+^ in the assay (Fig 3A). Turnover increases as the concentration of Mg^2+^ is increased, with highest turnover seen with an excess greater than calculated 4 Mg^2+^: 1 DHNTPase, perhaps due to the multitude of metal-binding species expected to exist in equilibrium in the assay mixture that compete for binding with DHNTPase.

To investigate stoichiometry of metal binding in solution and its effect on protein stability, ITC experiments and CD melts were conducted (Fig 3, Tables 2–4). Binding of Co^2+^ or Ni^2+^ to the DHNTPase in the presence of excess sulfate is entropically driven, likely due to the need to strip metals of their water ligands prior to complexation with the enzyme and sulfate anion. The positive enthalpy is attributable to water desolvation and the limited number of enthalpically-favorable protein-metal contacts. ITC data for metal binding were best fit to a model of independent binding sites of equal affinity (K_a_~1 x 10^5^ M^-1^) and a stoichiometry of 4, in support of crystallographic results (Tables 2 and 3, Fig 3B). There is no evidence for cooperativity in the binding reaction and full metal binding appears to be simultaneous as the data could not be fit satisfactorily to a sequential binding model (data not shown). Fully saturating the DHNTPase metal binding sites stabilizes the protein, as indicated by change in melting temperature (T_m_) from apo with sulfate (55.1 ± 0.3°C), to +4.2°C and +3.9°C when Co^2+^ or Ni^2+^ is added to the apo DHNTPase/sulfate solution (Table 4, Fig 3C). The addition of ligands does not interfere with the reversibility of the thermal melt (not shown).

**Table 2:** Thermodynamic parameters for metals binding to DHNTPase.

	Co^2+^	Ni^2+^
n	3.96 (0.07)	4.04 (0.06)
K_a_ (M^-1^ x 10^4^)	9.83 (0.21)	6.32 (0.11)
ΔH (kcal mol^-1^)	11.1 (0.6)	14.3 (0.4)
TΔS (kcal mol^-1^)	12.3 (0.4)	16.6 (0.3)
ΔG (kcal mol^-1^)	-1.2 (0.4)	-2.3 (0.3)

**Table 3:** Thermodynamic parameters for SO_4_^2-^ binding to DHNTPase.

	SO_4_^2-^
n	2.0
K_a1_ (M^-1^ x 10^4^)	11.2 (0.1)
ΔH_1_ (kcal mol^-1^)	5.7 (0.2)
TΔS_1_ (kcal mol^-1^)	11.7 (0.2)
ΔG_1_ (kcal mol^-1^)	- 6.0 (0.5)
K_a2_ (M^-1^ x 10^4^)	2.9 (0.2)
ΔH_2_ (kcal mol^-1^)	9.43 (0.24)
TΔS_2_ (kcal mol^-1^)	14.2 (0.3)
ΔG_2_ (kcal mol^-1^)	- 4.77 (0.37)

The value shown is the mean of three replicate experiments.

The number in parentheses is the standard deviation.

**Table 4:** Calculated T_m_ values for DHNTPase and DHNTPase-metal complexes from CD melt. The value shown is the mean of three replicate experiments. The number in parentheses is the standard deviation.

	Apo	SO_4_^2-^	Co^2+^/SO_4_^2-^	Ni^2+^/SO_4_^2-^
T_m_ (°C)	53.9 (0.3)	55.1 (0.4)	58.1 (0.2)	57.8 (0.3)

Notably, in the absence of metal, sulfate binding to apo DHNTPase is detectable, and associated with +1°C thermal stabilization (Table 4, Fig 3C). ITC data could only be fit by a sequential binding model with stoichiometry of n = 2 (Tables 2 and 3, Fig 3B), taking advantage of the *a priori* knowledge that two sulfates were observed crystallographically in the apo structure, one in the active site and the other on the surface ~ 20 Å away. We suggest that the higher Ka site corresponds to the active site sulfate, and may be a distorted binding site utilizing positively charged Arg29 and/or Lys7 in this region, and the second site is the one stabilized by Trp136 and Arg97 remote from the active site. The reasons sulfate binding to DNHTPase in the absence of divalent cations is not independent will require further investigation.

### Proposed substrate binding mode

**Fig 4:**
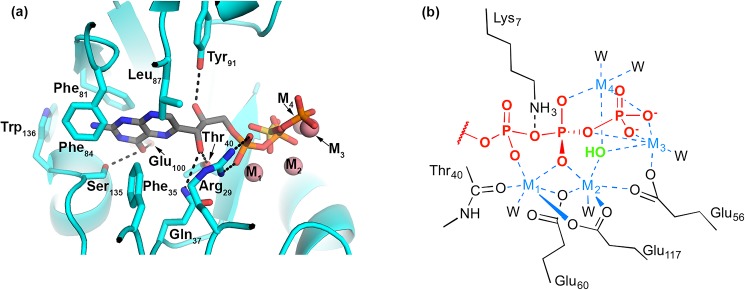
Substrate docking model. (a) Binding pocket for dihydroneopterin moiety with key predicted polar interactions presented in dashed lines and hydrophobic residues presented as sticks. Metals M1-4 are presented as balls but were not present during docking calculation. (b) Chemdraw representation of predicted interaction between triphosphate moiety with key stabilizing interactions with four metals and protein residues. Waters were excluded from docking calculation. Crystallographic water predicted to be activated for catalysis (green) is 2.6 Å from predicted scissile P-O bond in substrate. W = water.

*In silico* docking of the substrate DHNTP to apo DHNTPase (Fig 4A) followed by minor manual adjustment in the metal binding region (Fig 4B) reveals a binding pose compatible with the metallated structures, its bound sulfate anion, and the previous PPi-bound structures. The ring is nestled in a hydrophobic pocket lined by Phe35, Ile85, Phe84, and Leu87. Ser135 and Glu100 are predicted to stabilize the carbonyl oxygen of the ring, while Gln37, Arg29, Thr40, and Tyr91 stabilize the two hydroxyl groups (Fig 4A). The α-phosphate is stabilized by an apparent salt bridge with Arg29 and its coordination to M1; this phosphate is also within hydrogen bonding distance of Gln37, Thr40, and Lys7, further complementing the phosphate negative charge. The β-phosphate was docked in an orientation overlapping the crystallographic sulfate, where it is rendered electrophilic by coordinating to all four metal ions and forming a salt bridge with Lys7. The β-phosphate also overlays with PPi modeled in earlier structures, in which it overlaps with the more solvent exposed of the two phosphate groups (Figs 2D and 4A). In our model, M4, which does not have a protein-based ligand, coordinates the β- and γ-phosphates, activating the pyrophosphate leaving group.

The position of the dihydroneopterin ring in a distinct pocket within the active site of DHNTPase is compatible with a number of Nudix hydrolases cocrystallized with substrate, but our docking model differs from the one proposed previously in which the dihydroneopterin ring moiety was proposed to sit significantly deeper in the putative active site pocket [4], a pose we did not observe. The earlier docking model was inferred by presuming the crystallographic PPi represented the leaving group, e.g. the β- and γ-phosphates of the substrate, whereas our docking results indicate that the observed PPi more likely represents α- and β- positions of the substrate.

### Proposed catalytic mechanism

Nudix enzymes use nucleophilic substitution to hydrolyze their respective substrates, but literature reports vary in the residues involved in catalysis, and number and roles of divalent cations [6, 28]. Even studies of orthologs of the same enzyme, such as Ap4A [21, 26, 29], do not converge, leaving many open questions regarding the extent of mechanistic conservation across the Nudix family. In contrast to most available structures of Nudix hydrolases, the position and identity of four activating transition metal ions were enabled by our use of anomalous scattering data from transition metal ions that mimic Mg^2+^. Finally, binding was confirmed by ITC.

DHNTPase appears to function like Ap4A [21] and RppH [22], but with revisions to inferences from previous DHNTPase structures [4]. First, in line with observations for *C*. *elegans* Ap4A [21], a conformational change in the protein does not appear to be required for catalysis. Second, based on the fact that the Mg-soaked crystals contained only water, we infer that metal loading in DHNTPase is substrate-assisted, like other Mg-dependent enzymes involving nucleotide substrates [30, 31]. Third, consistent with our data and previous biochemical characterization of DHNTPase that identified a requirement for multiple metal ions and noted product inhibition [27], we propose that all four are required for catalysis and each plays a distinct role: M1 for positioning the α-phosphate, M2-M4 to activate the β-phosphate as well as the water for catalysis (see below), and M4 to further stabilize the pyrophosphate leaving group. At a free Mg^2+^ concentration of ~mM in *E*. *coli* [32], even for relatively weak binding affinity like Mg^2+^ for ATP [33], the metallated substrate predominates. We note that the observation of four metal ions in *C*. *elegans* Ap4A was unexpected at the time, and, while also observed in *P*. *falciparum* Ap4A [25], its physiological significance was uncertain given earlier biochemical studies using *B*. *bacilliformis* Ap4A [29] and structures solved subsequently from other organisms such as *C*. *trachomatis CT771* [26] that favor the requirement for three metal ions, equivalent to M1-M3 in our structures. Combined with the challenges encountered in attempts to obtain a substrate-analog bound structure and an apparent low intrinsic binding affinity of metal to DHNTPase, we raise the possibility M4, is in fact relevant to DHNTPase and possibly other Nudix hydrolases by activating the water for catalysis as well as stabilizing the pyrophosphate leaving group. In addition, pyrophosphate in the presence of excess metals can form inhibitory minerals that would occlude dihydroneopterin-containing molecules from the binding pocket.

Finally, related to the role of M4 is the outstanding question regarding the catalytic base in Nudix hydrolases. In *C*. *elegans* Ap4A and *E*. *coli* RppH the inferred catalytic base is Nudix signature residue Glu55 or Glu56 [21, 22] (Fig 2E). These residues are 3.8 or 3.5 Å from the metal-bridged solvent in those structures, respectively, similar to the 3.6 Å measured for Glu59 and the bridging solvent similarly positioned in DHNTPase. Alternative Glu residues have been proposed as the base in Ap4A orthologs [26, 29], but the case has also been made that a protein-derived general base is not necessary for divalent Nudix hydrolases given substantial pKa-lowering of the water by the metal ions in the cluster [34]. Indeed, on the basis of its level of sequence conservation and position in our structures in direct line with the apparent M2-M4-activated water and sulfur atom in the sulfate anion, deprotonation of the solvent in DHNTPase may not require a protein-based general base. Glu59 could participate in catalysis, perhaps by stabilizing an intermediate species or a transition state. Previous suggestions of Tyr91 or His118 for the catalytic base in DHNTPase [4], however, do not appear likely based on our substrate docking model. After nucleophilic attack of the activated water on the β-phosphate, negative charge will be compensated by M1 and M2, which are coordinated to one of the sulfate/phosphate oxygens, as well as Arg29 and Lys7, as previously proposed [4]. After the release of Mg-bound pyrophosphate, the dihydroneopterin monophosphate product, likely along with at least two cations, will follow, to enable the next catalytic cycle to proceed.

In sum, we used a combination of crystallographic, biochemical, and biophysical measurements, as well as *in silico* docking to identify a role for four metal ions in the catalytic process of the Nudix hydrolase DHNTPase. Three metal ions, M2-M4 play dual roles in stabilizing the substrate and activating a solvent molecule; the pKa lowering ability of Co^2+^ and Ni^2+^, and likely Mg^2+^, are sufficient to deprotonate the solvent for nucleophilic attack, but we identified Glu59 in a position that may serve as general base or transition state stabilizer. The fact that we were not successful in crystallographically trapping dihydroneopterin in the active site combined with the attractive docking pose obtained for the natural substrate DHNTP, we infer that an inhibitor containing a mimic of the mineral-like arrangement will be key to efficient inhibition of this enzyme.
